# Association of Serum Magnesium Concentration With Microvascular Complications Among Patients Diagnosed With Type 2 Diabetes Mellitus: An Observational Cross-Sectional Analysis

**DOI:** 10.7759/cureus.117070

**Published:** 2026-09-28

**Authors:** Sharwan Kumar, Sunil K Gothwal, Bhupendra Arya, Dilip Nagarwal, Chetram Kumawat, Anurag Mishra, Bhawana Sharma, Yogendra Singh, Aashutosh Sinwal, Gaurav Gupta

**Affiliations:** 1 Department of Medicine, Jawaharlal Nehru Medical College and Hospital, Ajmer, IND; 2 Department of Neurology, Jawaharlal Nehru Medical College and Hospital, Ajmer, IND; 3 Nims Institute of Pharmacy, Nims University Rajasthan, Jaipur, IND; 4 Department of Pharmaceutics, Maharishi Arvind College of Pharmacy, Jaipur, IND; 5 Department of Pharmacology, Maharishi Arvind College of Pharmacy, Jaipur, IND; 6 Department of Pharmacology, Centre for Research Impact &amp; Outcome-Chitkara College of Pharmacy, Chitkara University, Rajpura, IND

**Keywords:** diabetic nephropathy, diabetic neuropathy, diabetic retinopathy, glycemic control, hypomagnesemia, microvascular complications, serum mg, type 2 diabetic mellitus (t2dm)

## Abstract

Background

Persistent high blood sugar levels resulting from reduced insulin production, insulin resistance, or both are hallmarks of type 2 diabetes mellitus (T2DM). Magnesium (Mg) plays a role in insulin signaling and glucose metabolism. In individuals with T2DM, decreased Mg levels have been associated with decreased insulin function. The relationship between serum Mg levels and microvascular problems in individuals with T2DM was assessed in this study.

Materials and methods

We conducted an observational cross-sectional research involving 172 adults with T2DM attending the Medicine Department in Central Rajasthan. Serum Mg was measured using an automated clinical chemistry analyzer. Neuropathy was evaluated by history, neurological exam (deep tendon reflexes and monofilament testing), and nerve conduction studies if clinically indicated. Kidney function tests and urine examination were used to assess nephropathy, and ophthalmology referral for fundus examination was used to assess retinopathy. Unadjusted odds ratios (ORs) with Yates-corrected chi-squared testing were used to evaluate the association of the serum Mg status with each microvascular complication. Data were entered into Microsoft Excel (Microsoft Corp., Redmond, WA, USA) and analyzed using SPSS version 27 (IBM Corp., Armonk, NY, USA).

Results

Hypomagnesemia, defined as serum Mg < 1.7 mg/dL, was identified in 57 of 172 participants (33.14%), including 30 of 88 men (34.09%) and 27 of 84 women (32.14%). Compared with participants with Mg ≥ 1.7 mg/dL, those with hypomagnesemia had higher unadjusted odds of neuropathy (OR 7.10, 95% confidence interval (CI) 3.08-16.41), nephropathy (OR 4.73, 95% CI 2.06-10.84), and retinopathy (OR 9.35, 95% CI 4.49-19.47). They also had higher HbA1c (10.08% ± 2.07% vs 7.96% ± 1.74%; p < 0.0001) and longer diabetes duration (10.35 ± 5.95 vs 3.42 ± 3.16 years; p < 0.0001).

Conclusions

In this cohort of adults with T2DM, lower serum Mg levels were associated with poorer glycemic indices and a greater burden of microvascular complications. All ORs were unadjusted, and the groups of different serum Mg levels varied in terms of age, diabetes duration, and HbA1c, so the findings presented must be viewed as exploratory and may be affected by confounding.

## Introduction

Diabetes mellitus (DM) is a long-term metabolic disorder caused by decreased insulin secretion, action, or both, which keeps blood sugar levels above normal. Its prevalence has increased substantially over recent decades, creating a continuing need to address both glycemic control and diabetes-related complications. Type 2 DM (T2DM) accounts for the majority of diabetes cases worldwide. Earlier estimates projected that the global number of people with diabetes would reach 439 million by 2030 [1], while the burden in India was expected to represent a substantial proportion of this total [2].

Magnesium (Mg) is an essential mineral with a broad role in cellular physiology. It contributes to energy metabolism, nucleic acid stability, protein synthesis, and several enzyme-dependent reactions [3]. Low serum Mg has been reported in approximately 2.5%-15% of otherwise healthy populations and in approximately 14%-48% of people with diabetes [4]. In T2DM, Mg depletion has been associated with insulin resistance, inflammation, and oxidative stress, all of which may contribute to vascular injury [5]. Dietary intake is one determinant of Mg status, with foods such as legumes, dairy products, whole grains, green vegetables, and nuts providing important sources.

Mg also participates in insulin receptor signaling. Mg availability supports the kinase activity required after insulin binds to its receptor, thereby facilitating downstream signaling and glucose utilization [6,7]. Renal Mg handling is another important determinant of circulating Mg. Most filtered Mg is reabsorbed in the thick ascending limb, with additional recovery in the proximal and distal convoluted tubules [2]. Insulin influences renal Mg conservation in part through effects on the TRPM6 channel in the distal convoluted tubule [8,9]. In diabetes, osmotic diuresis associated with glycosuria can increase urinary Mg loss, while altered tubular function and oxidative stress may further impair Mg conservation [10-13].

These observations provide a biologically plausible link between Mg depletion and abnormal glucose metabolism. Reduced Mg availability may impair pancreatic β-cell function and insulin responsiveness, while poor metabolic control may itself promote urinary Mg wasting [14]. The resulting interaction could reinforce both Mg depletion and insulin resistance. Because chronic hyperglycemia is a major driver of diabetic microvascular injury, identifying whether low serum Mg is associated with neuropathy, nephropathy, and retinopathy may have clinical relevance.

Previous studies have compared the relationship between serum Mg, glycemic control, and insulin resistance in patients with T2DM; however, data from the studied population regarding the association between serum Mg status and individual DM microvascular complications are limited. This study provides complication-specific estimates from a hospital-based cohort in Central Rajasthan.

The main aim was to evaluate the association between hypomagnesemia (Mg < 1.7 mg/dL) and diabetic neuropathy, nephropathy, and retinopathy in adults with T2DM in comparison to those with ≥1.7 mg/dL of Mg. Estimation of the prevalence of hypomagnesemia and examination of its relationships with glycemic indices and other continuous clinical variables were secondary analyses.

## Materials and methods

We conducted a cross-sectional observational research in the outpatient department (OPD) and inpatient department of Medicine between October 2022 and September 2023. All patients diagnosed with T2DM who were visiting the Medicine OPD or were admitted to medical wards were screened according to the inclusion and exclusion criteria. Eligible patients were selected in a consecutive manner, and written informed consent was obtained. Inclusion criteria were a documented diagnosis of T2DM, age ≥ 18 years, and written informed consent. Patients receiving diuretics or those with non-diabetic chronic kidney disease (CKD), malabsorption syndrome, or persistent diarrhea were excluded from the study. Diabetic nephropathy was retained as a study outcome and therefore was not an exclusion criterion; it was defined according to the Kidney Disease: Improving Global Outcomes (KDIGO) 2022 criteria [15]. A total of 172 participants met the study criteria. The number of patients who were screened and subsequently excluded could not be reconstructed from the original records.

Sample size and data completeness

The sample size was calculated using the formula n = Z²pq/d². The expected proportion was 12% in light of the reported prevalence of T2DM in the Gwalior-Chambal region (11.4%) [16]. With p = 0.12, q = 0.88, an allowable error of 5%, and Z = 1.96 for a 95% confidence level, the minimum calculated sample size was 163. This calculation accounts for the prevalence of T2DM in the general population, which is not necessarily the prevalence of hypomagnesemia, and there was no prespecified microvascular association between the two. Recruitment was undertaken by screening all patients presenting during the recruitment period against the predefined criteria. A total of 172 patients were eligible and formed the final analytic group. The same core laboratory assessment was obtained for all participants. There were no withdrawals after enrolment, and no missing values were imputed.

Assessment of microvascular complications

Peripheral neuropathy was evaluated based on clinical history and neurological examination, which included deep tendon reflexes and monofilament testing. Patients who required additional tests were referred to the Department of Neurology for nerve conduction tests, where the diagnosis was made based on the neurological examination and the results of the nerve conduction tests. No numerical cut-off for nerve conduction velocity, latency, or amplitude was applied to the data, and no formal neuropathy grading or scoring system was used. The predefined exclusion criteria were CKD, malabsorption, alcoholic cirrhosis, malignancy, or any other specified systemic conditions that could lead to peripheral neuropathy or to an alteration in serum Mg.

Retinopathy was checked by referral for fundus examination to the Department of Ophthalmology. All patients referred by the ophthalmology team with diabetic retinopathy were considered diabetic retinopathy patients for this study. The investigators did not use a separate retinopathy grading system.

At the time of recruitment, patients with known and labeled CKD or acute kidney injury were excluded. Kidney function tests and urine examinations were performed for all enrolled participants. Renal involvement found in the evaluation was classified as a diabetic microvascular complication based on the protocol and KDIGO 2022 guidelines.

Laboratory assessment

Serum Mg concentration was determined with the study laboratory's automated clinical chemistry analyzer. Relevant study documentation was not available and could not be part of a retrospective reconstruction of the analyzer model, the name of the manufacturer of the reagents, and other assay quality-control information.

Statistical analysis

Data were entered into MS Excel (Microsoft Corp., Redmond, WA, USA) and analyzed with IBM SPSS Statistics 27 (IBM Corp., Armonk, NY, USA). Independent-samples t-tests were used to compare means and standard deviations of continuous variables, which are presented as mean ± standard deviation. Categorical data are displayed as numbers and percentages, and compared using chi-square with Yates correction as appropriate. Yates-corrected chi-squared testing and odds ratios (ORs) with 95% confidence intervals (CIs) were used to quantify the association between serum Mg status (<1.7 mg/dL vs ≥1.7 mg/dL) and each microvascular complication. All ORs are crude. No multivariable logistic regression was performed with regard to age, diabetes duration, and HbA1c. Pearson correlation and simple linear regression were used to assess the relationship between serum Mg and continuous clinical and biochemical variables. Receiver operating characteristic (ROC) curves were computed for specified variables, if applicable. Statistical significance was defined as a p-value of ≤0.05.

## Results

The study included 172 participants (88 men and 84 women). The ages ranged from 30 to 92 years, and the body mass index (BMI) ranged from 16.9 to 37.6 kg/m². The overall mean age was 56.40 ± 11.18 years, and the mean BMI was 26.47 ± 3.86 kg/m². The mean fasting glucose, postprandial glucose, and HbA1c levels were 137.13 ± 36.26 mg/dL, 244.50 ± 67.43 mg/dL, and 8.66% ± 2.21%, respectively. The mean serum Mg level was 1.80 ± 0.23 mg/dL. None of these variables differed significantly by sex (Tables 1, 2).

**Table 1:** Distribution of study variables (N = 172) Diabetes duration of 0 years represented patients diagnosed within the preceding year. SD: standard deviation; Mg: magnesium

Variable	Unit	Minimum	Maximum	Mean	SD
Age	years	30	92	56.40	11.18
Body mass index	kg/m²	16.90	37.60	26.47	3.86
Duration of diabetes	Years	0	30	5.72	5.39
HbA1c	%	5.50	15.00	8.66	2.21
Serum Mg	mg/dL	1.40	2.30	1.80	0.23
Fasting blood glucose	mg/dL	84	300	137.13	36.26
Postprandial blood glucose	mg/dL	135	485	244.50	67.43

**Table 2:** Distribution of study variables according to sex All comparisons used independent-samples t-tests with 170 degrees of freedom. None differed significantly by sex (all p > 0.05). SD: standard deviation; Mg: magnesium

Variable	Unit	Male (n = 88) mean	Male (n = 88) SD	Female (n = 84) mean	Female (n = 84) SD	t	df	p-value	Cohen's d
Age	years	56.18	10.95	56.62	11.47	-0.26	170	0.7972	-0.04
Body mass index	kg/m^2^	26.11	3.55	26.85	4.14	-1.26	170	0.2093	-0.19
Duration of diabetes	years	6.13	4.92	5.28	5.84	1.03	170	0.3026	0.16
HbA1c	%	8.81	2.24	8.51	2.18	0.89	170	0.3750	0.14
Serum Mg	mg/dL	1.78	0.22	1.81	0.25	-0.84	170	0.4041	-0.13
Fasting blood glucose	mg/dL	140.13	34.37	133.98	38.07	1.11	170	0.2673	0.17
Postprandial blood glucose	mg/dL	247.41	66.92	241.45	68.23	0.58	170	0.5638	0.09

When categorized by age, 62 participants (36.05%) were 30-50 years old, 94 (54.65%; 47 men and 47 women) were 51-70 years old, and 16 (9.30%) were >70 years old. Regarding BMI, 78 participants (45.35%) were in the 23-27.4 kg/m² range, 61 (35.47%) had a BMI ≥ 27.5 kg/m², 31 (18.02%) had a normal BMI, and two (1.16%) were underweight. The duration of diabetes was <5 years in 100 participants (58.14%), 5-10 years in 50 (29.07%), and >10 years in 22 (12.79%) participants.

Serum Mg was below 1.7 mg/dL in 57 participants (33.14%), whereas 113 (65.70%) were within the 1.7-2.2 mg/dL reference category and two (1.16%) had concentrations > 2.2 mg/dL (Table 3). For subsequent analyses, the 113 participants in the reference category and the two participants above the reference range were combined as the comparator group (n = 115; serum Mg ≥ 1.7 mg/dL).

**Table 3:** Serum Mg categories by sex Hypomagnesemia was defined a priori as serum Mg < 1.7 mg/dL. Mg: magnesium

Serum Mg category (mg/dL)	Male (n)	Female (n)	Total (n)	Percentage (%)
<1.7 (hypomagnesemia)	30	27	57	33.14
1.7-2.2 (reference range)	57	56	113	65.70
>2.2 (above reference range)	1	1	2	1.16
Total	88	84	172	100.00

Participants with hypomagnesemia (n = 57) had higher mean HbA1c, age, BMI, diabetes duration, fasting glucose, and postprandial glucose than those in the comparator group (n = 115). The mean HbA1c levels were 10.08% ± 2.07% versus 7.96% ± 1.74%; mean age was 61.19 ± 11.83 versus 54.02 ± 10.08 years; mean BMI was 27.53 ± 4.49 versus 25.95 ± 3.40 kg/m²; mean diabetes duration was 10.35 ± 5.95 versus 3.42 ± 3.16 years; mean fasting glucose was 146.47 ± 29.41 versus 132.50 ± 38.49 mg/dL; and mean postprandial glucose was 274.37 ± 58.15 versus 229.69 ± 67.03 mg/dL. The corresponding t-test results were significant for all six comparisons: HbA1c, t(170) = 7.05, p < 0.0001, d = 1.14; age, t(170) = 4.14, p < 0.0001, d = 0.67; BMI, t(170) = 2.57, p = 0.0110, d = 0.42; diabetes duration, t(170) = 9.98, p < 0.0001, d = 1.62; fasting glucose, t(170) = 2.41, p = 0.0169, d = 0.39; and postprandial glucose, t(170) = 4.29, p < 0.0001, d = 0.70 (Table 4).

**Table 4:** Clinical and glycemic characteristics according to serum Mg status Independent-samples t-test; df = 170 for each comparison, with statistical significance defined as p ≤ 0.05. The comparator group (Mg ≥ 1.7 mg/dL) included 113 participants in the reference range and two participants above the reference range. Mg: magnesium; SD: standard deviation

Parameter	Unit	Hypomagnesemia < 1.7 mg/dL (n = 57) mean	Hypomagnesemia < 1.7 mg/dL (n = 57) SD	Normomagnesemia ≥ 1.7 mg/dL (n = 115) mean	Normomagnesemia ≥ 1.7 mg/dL (n = 115) SD	t	df	p-value	Cohen's d
HbA1c	%	10.08	2.07	7.96	1.74	7.05	170	<0.0001	1.14
Age	years	61.19	11.83	54.02	10.08	4.14	170	<0.0001	0.67
Body mass index	kg/m²	27.53	4.49	25.95	3.40	2.57	170	0.0110	0.42
Duration of diabetes	years	10.35	5.95	3.42	3.16	9.98	170	<0.0001	1.62
Fasting blood glucose	mg/dL	146.47	29.41	132.50	38.49	2.41	170	0.0169	0.39
Postprandial blood glucose	mg/dL	274.37	58.15	229.69	67.03	4.29	170	<0.0001	0.70

Neuropathy was identified in 33 participants (19.19%), nephropathy in 30 (17.44%), and retinopathy in 56 (32.56%). Twenty-nine participants had at least two microvascular complications, and nine had all three. Compared with participants whose serum Mg was ≥1.7 mg/dL, those with Mg < 1.7 mg/dL had higher unadjusted odds of neuropathy (OR 7.10, 95% CI 3.08-16.41), nephropathy (OR 4.73, 95% CI 2.06-10.84), and retinopathy (OR 9.35, 95% CI 4.49-19.47). The corresponding Yates-corrected chi-squared tests were significant for neuropathy (χ²(1, N = 172) = 22.631, p < 0.0001, φ = 0.378), nephropathy (χ²(1, N = 172) = 13.346, p = 0.0003, φ = 0.295), and retinopathy (χ²(1, N = 172) = 38.468, p < 0.0001, φ = 0.486). The serum Mg groups were not matched for age, duration of diabetes, or HbA1c, so there could be confounding factors affecting these unadjusted estimates (Table 5; Figure 1).

**Table 5:** Unadjusted odds ratios of microvascular complications associated with hypomagnesemia. Adjustment for age, diabetes duration, and HbA1c was not performed CI: confidence interval; Mg: magnesium

Complication	Total cases (n)	Low Mg, exposed (n = 57)	Normal Mg, non-exposed (n = 115)	Odds ratio	95% CI lower	95% CI upper	Yates-corrected chi-square	df	N	p-value	Phi
Neuropathy	33	23	10	7.10	3.08	16.41	22.631	1	172	<0.0001	0.378
Nephropathy	30	19	11	4.73	2.06	10.84	13.346	1	172	0.0003	0.295
Retinopathy	56	37	19	9.35	4.49	19.47	38.468	1	172	<0.0001	0.486

**Figure 1:**
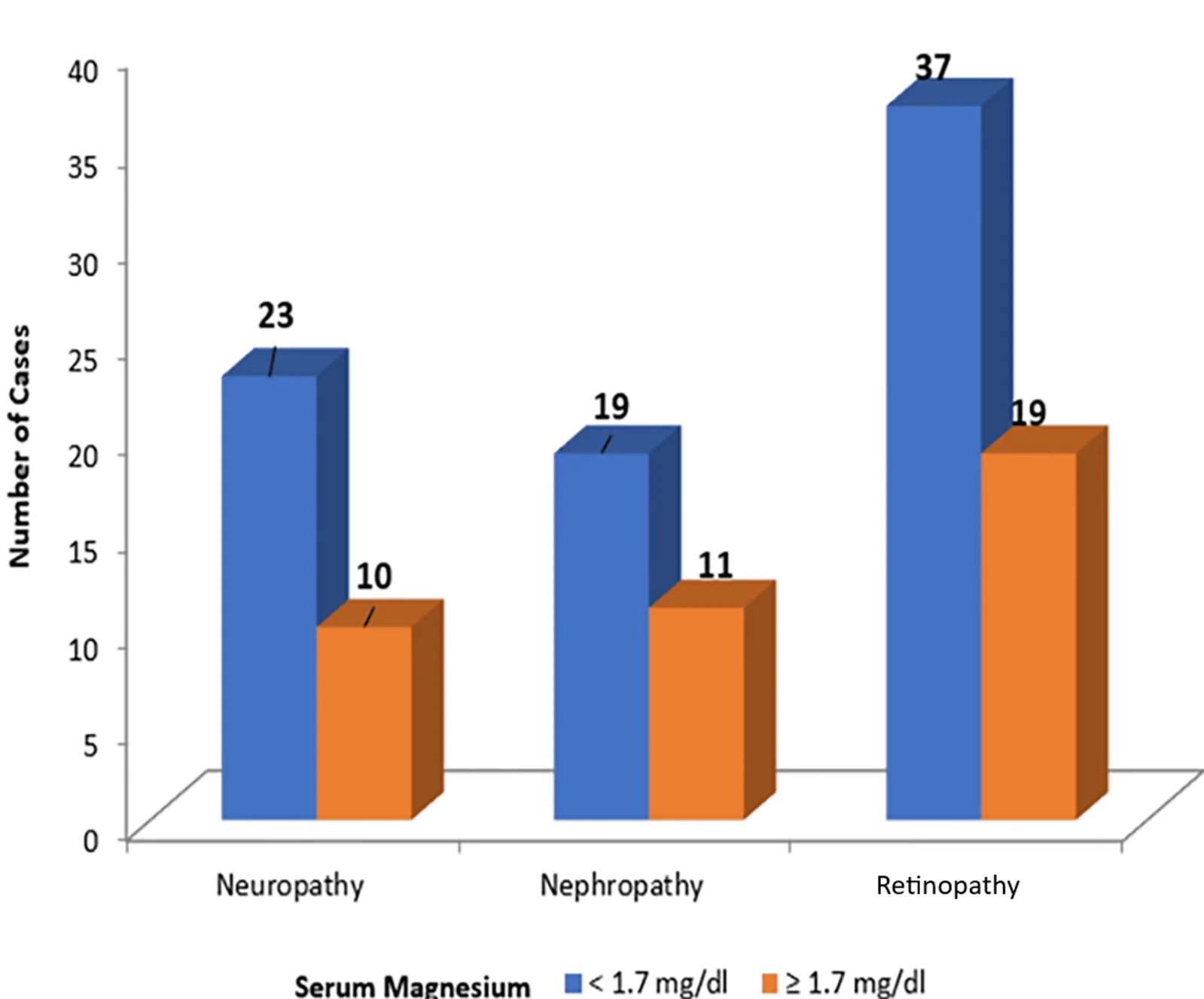
Number of neuropathy, nephropathy, and retinopathy cases according to serum magnesium (Mg) category in patients with type 2 diabetes mellitus (T2DM)

## Discussion

The prevalence of hypomagnesemia in this cohort was 33.14% (57/172). This estimate is close to the approximately 32% pooled prevalence reported in a recent systematic review and meta-analysis of observational studies in T2DM [17]. Other hospital-based studies have reported different frequencies. Moradiya and Muley identified serum Mg < 1.8 mg/dL in 37.68% of their participants [18], while Kumar et al. reported a prevalence of 44% in a New Delhi cohort [19]. Direct comparison should be made cautiously because the diagnostic thresholds, participant characteristics, and clinical settings were not identical.

The biological relationship between diabetes and low Mg levels is likely multifactorial. Potential contributors include reduced gastrointestinal absorption, insulin-related intracellular shifts, and increased renal Mg losses accompanying glycosuria. Diabetes-related tubular dysfunction may further alter Mg handling. Experimental and clinical evidence indicates that Mg availability influences insulin signaling and cellular glucose uptake, providing a plausible mechanism for the association observed in the present study [14].

In our cohort, hypomagnesemia was accompanied by less favorable glycemic and clinical characteristics. Participants with Mg < 1.7 mg/dL had higher HbA1c, age, BMI, fasting glucose, postprandial glucose, and diabetes duration than those with Mg ≥ 1.7 mg/dL. Moradiya and Muley observed hypomagnesemia in 78% of patients with HbA1c levels > 7% [18]. Kumar et al. reported relationships between low Mg and BMI, fasting glucose, postprandial glucose, and HbA1c [19], while Yossef et al. found higher glucose and HbA1c levels among patients with hypomagnesemia [20]. Taken together, these observations support an association between low Mg status and poor metabolic control, although the direction of this relationship cannot be established from cross-sectional data.

All three microvascular outcomes examined were associated with hypomagnesemia. The strength of association was greatest for retinopathy (OR 9.35), followed by neuropathy (OR 7.10) and nephropathy (OR 4.73). Previous studies have likewise described associations between low Mg and diabetic microvascular disease. Moradiya and Muley reported associations with non-proliferative retinopathy and nephropathy [18]. Kumar et al. found substantial frequencies of both non-proliferative and proliferative retinopathy among hypomagnesemic participants [19], and Dasgupta et al. reported a greater burden of retinopathy in patients with low Mg [21]. Differences in effect estimates across studies may reflect variations in sample size, population characteristics, Mg thresholds, and methods used to define complications.

Reported ORs have not been adjusted for age, diabetes duration, or HbA1c. It may be that these characteristics accounted for some of the observed associations, as they varied significantly across the serum Mg groups. Thus, the estimates should be interpreted as an unadjusted association and not independent effects of serum Mg. The present findings should be interpreted as evidence of an association rather than causation. Because exposure and outcomes were assessed within a cross-sectional framework, it was not possible to determine whether Mg depletion contributes to microvascular injury, whether advanced diabetes promotes Mg loss, or whether both processes coexist. Longitudinal studies and randomized intervention trials are needed to determine whether correcting Mg deficiency changes glycemic control or alters the incidence or progression of diabetic microvascular complications.

Limitations

Several limitations should be considered. First, the cross-sectional design prevented the assessment of temporal sequence and causal effects. Second, information on dietary Mg intake, Mg supplementation, and medication adherence was not collected, leaving potential residual confounding. Excluding diuretic users reduced one important source of Mg-related confounding. Third, although the sample size exceeded the calculated minimum (172 vs 163), the study may have had limited power to detect smaller effects, particularly for less frequent outcomes. Fourth, recruitment at a single center may have limited generalizability and introduced the possibility of selection bias. The use of one standardized analyzer, however, reduced interlaboratory measurement variability. Fifth, fasting and postprandial glucose were measured using a capillary glucometer rather than a venous laboratory assay. Sixth, the ORs were not adjusted; the differences between the groups may partly explain the observed ORs, as they were not adjusted for differences in age, diabetes duration, and HbA1c levels. Seventh, the definitions for complications were based on clinical practice, which means that uniform and investigator-defined grading systems were not used; this introduces the potential for outcome misclassification. Eighth, the total number of patients who were screened and excluded or full technical details of the serum Mg assay were not reported. Finally, serum Mg was measured once, so within-person biological variation and preanalytical factors were not separately assessed.

## Conclusions

In this hospital-based cohort of adults with T2DM, approximately one-third had serum Mg levels below 1.7 mg/dL. Hypomagnesemia was associated with higher HbA1c, BMI, fasting and postprandial glucose, and longer diabetes duration. It was also associated with substantially greater unadjusted odds of neuropathy, nephropathy, and retinopathy. Since these estimates were not adjusted for age, duration of diabetes, or HbA1c, and the study was cross-sectional, these should only be regarded as exploratory associations and not necessarily evidence of an independent effect of serum Mg. The results do not indicate that Mg deficiency leads to microvascular disease or that Mg supplementation affects microvascular disease. Until there are some prospective studies and controlled supplementation trials, supplementation cannot be recommended for this purpose.
